# Supplementary material for: An implantable, wireless, battery-free system for tactile pressure sensing
Source: Microsyst Nanoeng. 2023 Oct 11;9:130. doi: 10.1038/s41378-023-00602-3 (PMC10564885; doi:10.1038/s41378-023-00602-3)
Supplement: Supplementary file 1 — Supplementary Materials [file 41378_2023_602_MOESM1_ESM.docx]

**An implantable, wireless, battery-free system for tactile pressure sensing**

**SUPPLEMENTARY MATERIALS**


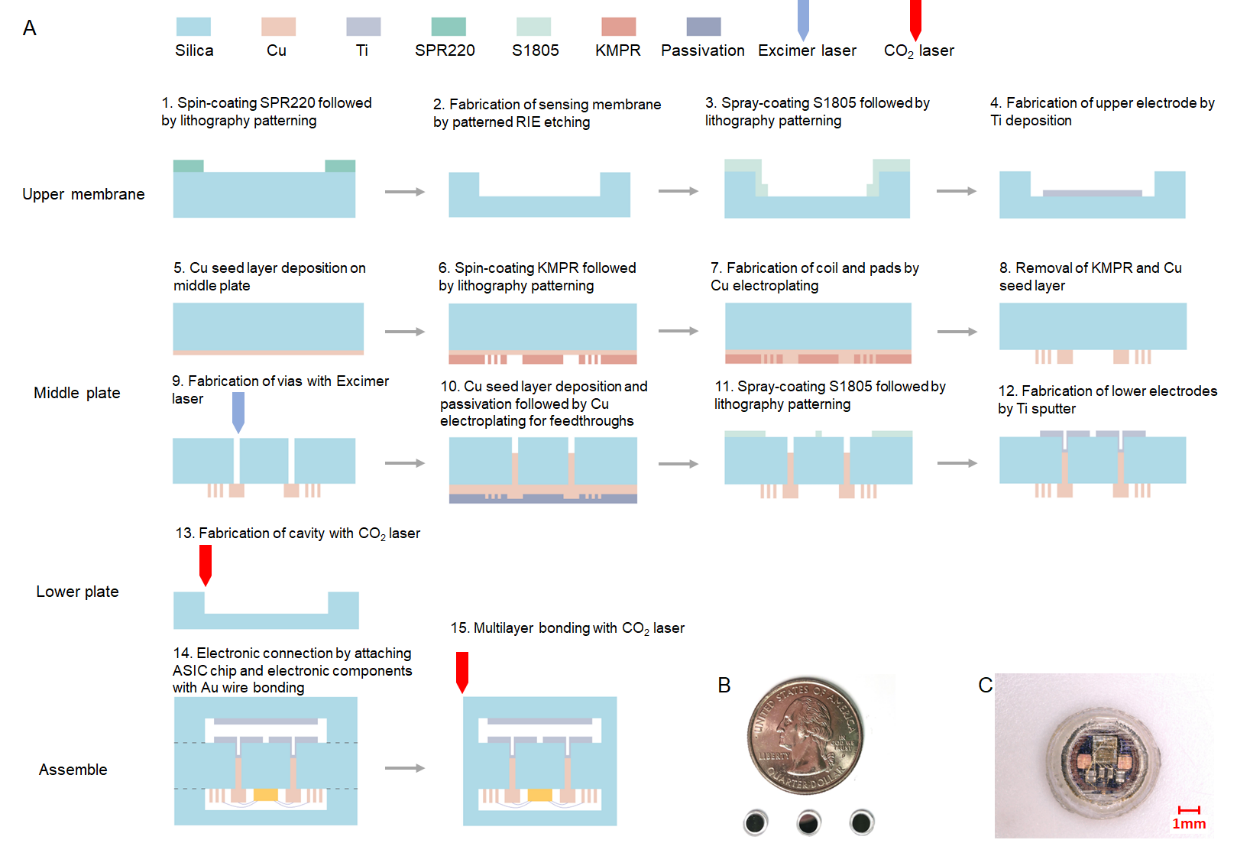


**Figure S1. Schematic illustrations for fabricating and assembling the tactile sensing system.** (**A**) **Fabrication of the upper plate**: 1. Spin coating SPR220 on silica substrate and perform photolithography. 2. Fabrication of sensing membrane cavity by RIE etching. 3. Spray coating S1805 on silica substrate with cavity, followed by photolithography. 4. Fabrication of upper electrode by depositing Ti. **Fabrication of the middle plate**: 5. Deposit Cu seed layer on silica middle plate. 6. Spin coating KMPR on Cu seed layer and perform photolithography. 7. Fabrication of coil and pads by Cu electroplating. 8. Remove KMPR and Cu seed layer. 9. Fabrication of vias by Excimer laser rastering. 10. Deposit Cu seed layer and passivate the seed layer to electroplate Cu feedthroughs. 11. Spray coating S1805 and perform photolithography on the other side of the middle plate. 12. Fabrication of the lower electrodes and connection to underneath feedthroughs by sputtering Ti. **Fabrication of the lower plate**: 13. CO_2_ laser rastering. **Assembly of electronics and device:** 14. Attach ASIC chip and electronic components on the middle plate and use Au wire bond to establish electrical connection. 15. CO_2_ laser multilayer fusion bonding. (**B**) Top view of three sensor prototypes compared with a U.S. quarter dollar. (**C**) bottom view of the sensing system.


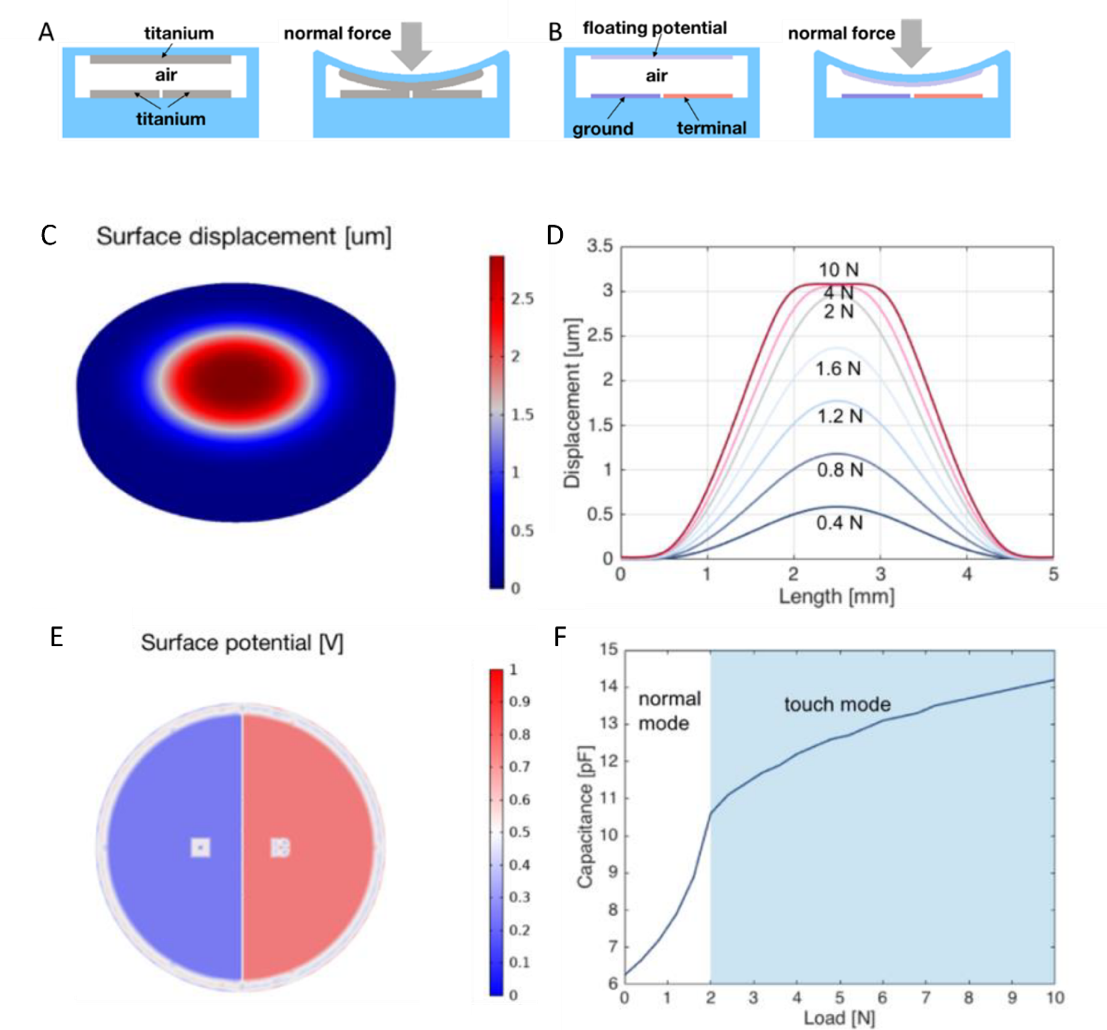


**Figure S2. COMSOL simulation of the capacitive sensor.** To investigate the sensor behavior under an applied normal force, a COMSOL multiphysics simulation was performed. The loading procedure was simulated in a two-step fashion. (**A**) In the first step, the solid mechanics module was applied to study the deflection of the upper plate. When there was no force applied, the upper plate and the lower substrate were parallel. Since the inward-facing surfaces of the sensor bear electrodes, the initial configuration of the electrodes was also parallel. The space between the electrodes was modelled as air. The electrodes were made from Ti and a thin layer of silicon dioxide (SiO_2_) at the surface of the electrodes. When there was normal force applied to the upper plate, the resultant deflection reduced the air gap until the two plates touched. Once the touch point was established, regions of the respective electrode layers were touching with no air gap between them. The insulating nature of the oxidation layer prevented top-to-bottom electrode shorting. (**C,D**) The deflection of the sensor when 10 N was applied over a 1.5 mm region coaxial with the center of the sensor. As expected, the central region of the upper plate experienced maximal deflection, and the peripheral region of the upper plate (which is bonded to the substrate) experienced minimal deflection. From 0-2 N, the deflection of the upper plate increased gradually, indicating that the sensor was operating in normal mode. Above 2 N, the maximum deflection remained almost constant, indicating touch mode operation. As larger forces were applied, the sensor continued to operate in touch mode as larger areas came into contact. (**B**) For the second step, the solid mechanics and AC/DC electrostatics modules were used to determine the sensor capacitance for each deflection profile. A floating potential was assigned to the top electrode. (**E**) The lower right electrode was designated as the terminal electrode with a 1V potential and the lower left electrode was designated as ground. (**F**) The capacitance of the sensor under each deflection profile could then be obtained from this electrostatics study. From 0-2 N, the sensor operated at normal mode. At 2 N, contact between the two plates occured and touch mode began. Above 2 N, the sensor continued to operate in touch mode and the capacitance increased more slowly.


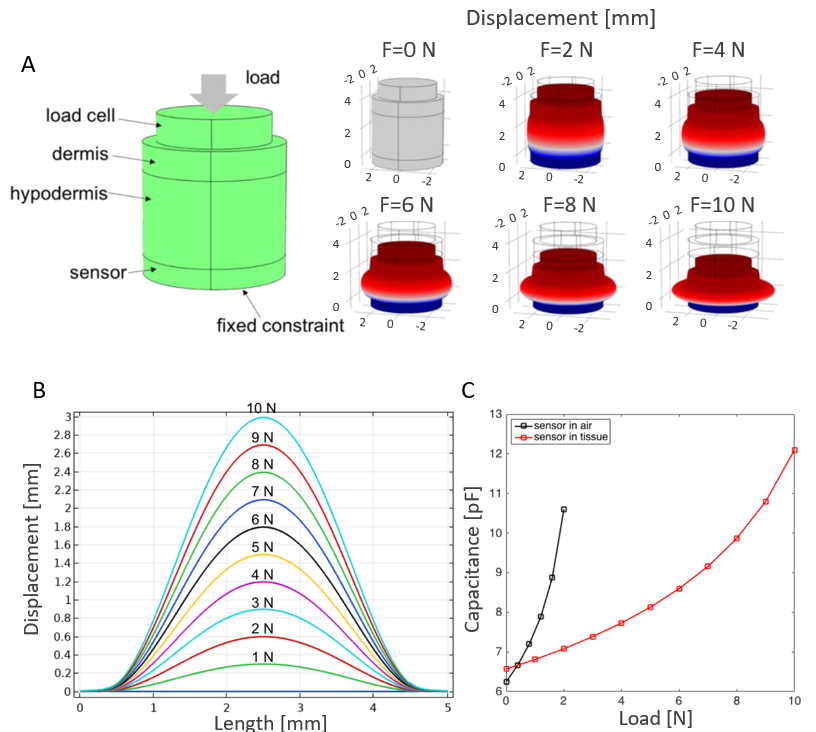


**Figure S3. COMSOL simulation of the capacitive sensor implanted under skin. (A)** A 1-mm thick dermis layer with Young’s modulus of 1000 kPa and Poisson’s ratio of 0.48 as well as a 3-mm thick hypodermis layer with Young’s modulus of 50 kPa and Poisson’s ratio of 0.48 were built on top of the sensor. Normal force was applied on top of the dermis layer with a 4 mm diameter stainless steel load cell and the bottom surface of the sensor was fixed. Tissue deformations shown as force was increased from 0 to 10 N. Due to the low Young’s modulus of the hypodermis layer, the deformation of the hypodermis was more significant than the deformation of the dermis layer. (**B**) The deflections of the sensor upper plate in response to 0 to 10N applied forces are shown. (**C**) In step 2 of simulation, the simulation model was surrounded by an environment with permittivity of 80 to simulate the extracellular fluid environment. The capacitance of the sensor with dermis and hypodermis layers was equivalent to 23% direct force on the sensor, indicating that a 10 N force applied to the skin surface was equivalent to a 2.3 N force applied directly on the upper plate of the implanted sensor. The unloaded (0N) output capacitance increased 0.35 pF in the implanted sensor simulation compared to the direct force simulation due to high permittivity environment of the former.

**
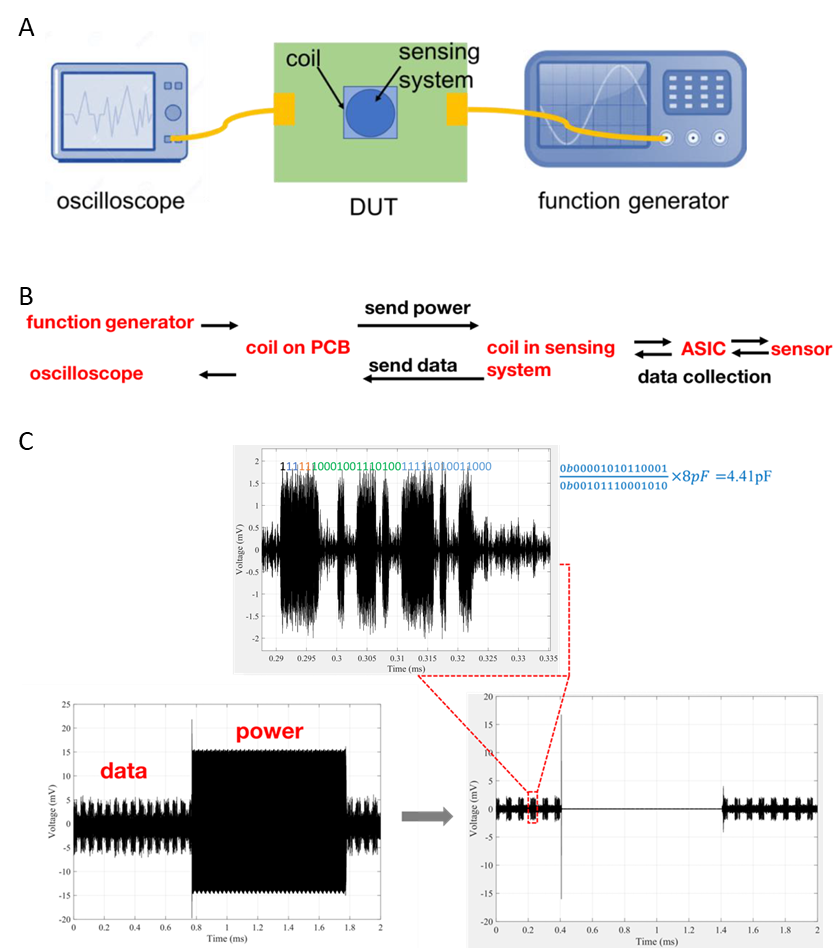
**

**Figure S4. Wireless communication between the sensing system and the base unit. (A)** To characterize the tactile sensing system wirelessly, a customized experimental setup. The base unit comprised a customized device under test (DUT) including a coil, electronic components, and two SMA connectors. One connector on the PCB was connected to a function generator and the other was connected to an oscilloscope. The tactile sensing system prototype was placed on top of the coil on the DUT. (**B**) During the measurement, the function generator formed a sinusoidal wave that was connected to the coil on PCB. This signal sent power to the coil in the sensing system wirelessly. Then, the power received by the coil in the sensing system provided energy for the ASIC which collected capacitive sensing data. Using a time multiplexing method, the function generator was then turned off. The data collected from the sensor was sent out through the coil in the sensing system. This data was received by the coil on the PCB and read out by the oscilloscope. (**C**) The power signal and the sensing data signal received by the oscilloscope were saved for processing. In this example, the periodic signals from 0 - 0.8 seconds indicate data signals and the signals from 0.8 -1.8 second with higher amplitude indicate power signals. First, the power signal was eliminated so that sensing data could be taken for further analysis. Next, digital information was recognized from the signal and transformed to the capacitance value.
